# The implementation of a HACCP system improved the efficiency of a bull semen collection and processing center

**DOI:** 10.21451/1984-3143-AR2017-022

**Published:** 2018-08-16

**Authors:** Karina Lemos Goularte, Carlos Eduardo Ranquetat Ferreira, Elisângela Mirapalheta Madeira, Eduarda Hallal Duval, Arnaldo Diniz Vieira, Rafael Gianella Mondadori, Thomaz Lucia

**Affiliations:** 1 ReproPel, Faculdade de Veterinária, Universidade Federal de Pelotas, Pelotas, RS, Brazil.; 2 Faculdade de Veterinária, Universidade Federal de Pelotas, Pelotas, RS, Brazil.; 3 Departamento de Veterinária Preventiva, Laboratório de Inspeção de Produtos de Origem Animal (LIPOA/UFPel), Faculdade de Veterinária, Universidade Federal de Pelotas, Pelotas, Brazil.; 4 Departamento de Morfologia, Instituto de Biologia, UFPel, Pelotas, Brazil.

**Keywords:** bull sperm, HACCP, microbial counts, quality control, semen quality

## Abstract

Bull Semen Collection and Processing Centers (SCPC) have satisfactory control of sperm quality, but commonly lack standardized quality control of hygiene procedures. This study assessed the impact of implementing a Hazard Analysis and Critical Control Points (HACCP) system in a bull SCPC, comparing microbial counts on various steps of semen processing, semen quality and costs across two periods (before and after the HACCP implementation). After surveying all routine activities of the SCPC, control points were identified, preventive measures were designed and corrective actions were employed, whenever necessary. Six months after HACCP implementation, the system was audited and production data covering two similar periods of two consecutive years were compared. Counts of colony forming units in samples collected from artificial vaginas, flexible tubes from the straw filling machine and from fresh and frozen semen after HACCP implementation were lower than during the previous period (P < 0.05). Improved post-thawing sperm motility, membrane integrity and acrosome integrity (P < 0.0001) and reduced rejection of semen batches and frozen doses were observed after HACCP implementation (P < 0.01), resulting in reduced opportunity costs. Thus, the implementation of a HACCP system in a bull SCPC allowed low-cost production of high-quality semen doses with reduced microbial contamination.

## Introduction

The income from production and commercialization of bull semen doses is constantly increasing around the world (Associação Brasileira de Inseminação Artificial - ASBIA, 2016; National Association of Animal Breeders - NAAB, 2017 ). Bull semen production centers (SCPC) should have thorough quality control to supply their customers with semen doses with the highest possible quality, since many sources of variation (physiological, methodological and operational) may influence the final quality of their product ( Alvarez *et al*., 2005 ). Even though assessment of semen quality is usually efficient in bull SCPC, control procedures concerning hygiene during semen collection and processing are scarcely effective.

Hazard analysis and critical control points (HACCP) concepts are applied as quality control systems, using field data to identify critical hazards, generating and testing risk management strategies ( Hulebak and Schlosser, 2002 ) and establishing preventive measures that are usually more efficient than corrective measures ( Codex Committee on Food Hygiene, 2009 ). Although such decision-support systems were primarily applied for animal health management ( Rose *et al*., 2003 ; Bell *et al*., 2009 ; Horchner and Pointon, 2011 ) and in the food industry ( Ropkins and Beck, 2000 ; Lupin *et al*., 2010 ; Wang et *al*., 2010 ), strategies based on HACCP concepts have been also applied in other fields.

Instructions about procedures to be conducted during sperm collection and handling are provided by the terrestrial animal health code (World Organization for Animal Health - OIE, 2014). Nevertheless, bull SCPC currently have no standardized methodology guiding hygiene procedures. Quality control measures based on HACCP principles have been used to benchmark distinct boar SCPC ( Schulze *et al*., 2015 ). Additionally, a HACCP system was recently developed for bull SCPC, identifying microbiological, physical and chemical hazards and determining three critical control points ( Goularte *et al*., 2015 ). However, the impact of the implementation of such a system on the efficiency of a bull SCPC on producing high-quality semen doses is not yet reported. The objective of this study was to compare parameters of sperm quality and of microbiological contamination and estimated costs and financial losses during the production of semen doses, before and after the implementation of a HACCP system in a bull SCPC.

## Material and Methods

### Data collection and documentation

The HACCP system was planned to enhance the final quality of the frozen semen doses produced in a bull SCPC by systematizing the production process ( Goularte *et al*., 2015 ), based on principles described elsewhere ( Hulebak and Schlosser, 2002 ). The HACCP system was implemented in a SCPC of a leading international industry that is certified by the Brazilian Ministério da Agricultura, Pecuária e e Abastecimento (MAPA) to produce and market bull semen doses worldwide. That SCPC is located in Delta, MG, Brazil (19º58'36” S; 47º46'16” W), housing nearly 120 bulls and processing in average 1.5 million frozen semen doses annually. The bulls remained at the SCPC during a period necessary to produce enough semen doses to supply the market or for private use, but no relevant changes in bull inventory occurred during the implementation of the HACCP program. All routine activities of the SCPC were monitored during thirty days, from initial steps such as the evaluation of the health status of the bulls and teaser cows and the hygiene of the bulls’ prepuce prior to semen collection up to the release of semen doses for sale. All procedures were detailed in manuals for Good Processing Practices (GPP), Standard Operating Procedures (SOP) and Standard Sanitizing Operating Procedures (SSOP), which were the documents used to apply for intellectual property protection of that HACCP system ( Goularte *et al*., 2011 ). Briefly, all bulls that arrived at the SCPC were quarantined, dewormed, vaccinated and routinely submitted to serological monitoring, as recommended by MAPA. When a health problem was detected in a breeder, that bull was excluded from semen collection. At the SCPC, bulls were maintained in pasture. Subsequently to the implementation of the HACCP system, the working routine of the SCPC was updated following the guidelines recorded in the manuals referred above, to keep the continuous training of all employees of the SCPC and to periodically audit their activities. Therefore, prior to the implementation of the HACCP principles, the production system of that SCPC could be considered unstandardized and after the HACCP implementation that system could be considered standardized.

### Semen processing and evaluation

Semen was collected with a sanitized and disinfected artificial vagina. All components of the artificial vagina were washed with a neutral detergent and rinsed under running water. Thereafter, the hard cylinder was kept at room temperature do dry. Rubber lines and cones were immersed in kilol solution (1:250) during 15 min, subsequently rinsed under distilled water and kept in a dryer for 30 min. Sperm morphology was evaluated every two weeks for each bull, however bulls that presented high percent of sperm pathologies at previous collections were evaluated weekly ( Goularte *et al*., 2015 ). Semen samples were frozen in egg yolk-sodium citrate (EYC) extender according to Shoae and Zamiri (2008) with little modifications as follows: 7% glycerol (v/v); 2.9% sodium citrate (w/v) buffer in autoclaved distillated water; 20% egg yolk (v/v); 50 µg/ml tylosin; 250 µg/ml gentamicin; and 150/300 μg/ml lincomycin-spectinomycin. The buffers were filtered before use. Initially, semen samples were diluted 1:1 (v/v) at 35°C in a pre-dilution extender, with the same composition mentioned above, but without glycerol. Then, semen samples were conditioned at 4◦C in a step-1 freezing extender (without glycerol) and subsequently in a step-2 freezing extender containing 14% glycerol ( Shoae and Zamiri, 2008 ). The cooling and freezing curves were as follows: 1 h at 9°C; 2 h at 4°C; final dilution for 1h at 4°C, when both freezing extenders were added; and freezing using an automated device (Digitcool 5300® CE ZH 350 & UE 350 with 900 HP Programmer, IMV Technologies, France). Both freezing extenders were previously stored at 5◦C for no longer than 72 h (OIE, 2014).

Frozen semen samples collected before the HACCP implementation (n = 35) and eight months after its implementation (n = 21) were transported to the ReproPEL laboratory, where they were processed by the same trained technician. Straws were thawed in a water bath at 37^o^C during 30 sec. Sperm motility was evaluated using optical microscopy at 200X. The integrity of the sperm membrane and the acrosomal status were both evaluated using an epifluorescent microscope (Olympus® BX 51) at 400X, with 450-520 nm filter wave length, counting 200 spermatozoa per samples. Sperm membrane integrity was evaluated using carboxyfluorescein diacetate and propidium iodide. Cells with intact membrane presented green fluorescence, whereas those with damaged membrane presented either red or simultaneous red and green fluorescence ( Harrison and Vickers, 1990 ). Acrosome integrity was evaluated using propidium iodide and *Lectin pisum sativum *, staining acrosomes with green fluorescence ( Kawamoto *et al*., 1999 ). Intact acrosome presented normal conformation, whereas damaged acrosome either were not evident or presented abnormal conformation.

### Microbiological analyses

To evaluate potential contamination at points considered hazardous for the quality of the semen doses, samples were collected from: artificial vaginas; fresh semen; pre-dilution extender; step-1 and step-2 freezing extenders; cooled semen; flexible tube from the filling machine (FT); packed semen; and thawed semen. The artificial vaginas and the FT were washed with 10 ml of autoclaved buffered peptone water (BPW). Samples from extenders, semen and washed from artificial vaginas and FT were serially diluted in BPW or seeded pure, as necessary. Cultures were performed in duplicate using 500 μl of each sample on plate count agar (PCA), as recommended by the World Organization for Animal Health, seeded by the Plour Plate method and incubated at 37°C for 72 h. Thereafter, plates having less than 300 colonies were counted, since greater number of colonies could not be reliably counted. The results were expressed as colony forming units (CFU/ml). Serial dilution was done, whenever necessary. Eight months after the implementation of the HACCP system, samples from the same points were collected and cultured again, to be compared with those collected previously.

### Efficiency of the HACCP system

Monitoring was conducted during the first 30-days period after HACCP implementation, as detailed by Goularte *et al*. (2015) . Based on the monitored control points, corrective actions were designed and executed, whenever occurred a deviation on the recommended guidelines. Preventive measures were implemented for the control points identified during monitoring. An audit was conducted six months after the HACCP implementation, to check whether routine procedures were executed as required.

The number of collected ejaculates, of processed and rejected semen batches and semen doses were collected for the same four month-period (October, November, December and January) of two consecutive years, with the objective of preventing relevant seasonal and environmental effects on the collected data. According to the criteria routinely used in the SCPC, semen samples were rejected when sperm motility was inferior to 30% after thawing and lower than 15% after a 4 h thermal stress test. In such cases, the entire semen batch was rejected. The economic impact of the HACCP implementation was estimated based on an average cost of US$1.00 per dose produced, including fixed and variable costs (unpublished data provided by the SCPC, 2011, Delta, MG, Brazil). The opportunity cost, defined as the cost of a delay on obtaining revenues after any key event ( Polson *et al*., 1993 ), was based on an average sales price of US$ 10.00 per semen dose.

### Statistical analysis

Due to lack of normality detected by Shapiro-Wilk test, the number of CFU/ml at distinct collection points was transformed to the logarithmic scale and subsequently compared between the periods before and after the HACCP implementation using analysis of variance, with comparison of means by the Tukey® test. Post-thawing sperm motility, membrane integrity and acrosome integrity were compared between periods using the Wilcoxon ranks sum test for nonparametric data, also due to lack of normality. The percent of rejected semen batches and doses were compared between periods using the chi-square test. All analyzes were conducted with Statistix® (2013) .

## Results

After the implementation of the HACCP system, microbial counts were lower than during the previous period (P < 0.05), in samples from artificial vaginas, FT, fresh semen and frozen semen ( Table 1 ). No difference in microbial counts was observed for samples of packed semen (P > 0.05). However, the number of CFU/ml in cooled semen samples was increased after the HACCP implementation (P < 0.05).

**Table 1:** Microbial counts (x 10^3^ CFU/ml) in distinct collection points in a bull semen production center, before and after eight months of the implementation of a HACCP system.

Sample	n	Pre-implementation	n	Post-implementation
Artificial vagina	13	0.081 ± 0,019^a^	10	0.019 ± 0.019^b^
Fresh semen	35	41.8 ± 0.019^a^	21	9.8 ± 4.1^b^
Pre-dilution extender	10	0.115 ± 0.006	9	1.9 ± 0.008
Step-1 freezing extender	10	0.056 ± 0.003	9	2.4 ± 1.0
Step-2 freezing extender	10	0.078 ± 0.006	9	0.873 ± 0.006
Cooled semen	35	0.240 ± 0.010^a^	21	1.5 ± 0.005^b^
Flexible tube from filling machine	21	2,500 ± 5.200^a^	15	0^b^
Packed semen	35	1,200 ± 66	21	1.7 ± 0.004
Frozen semen	35	23.3 ± 4.1^a^	21	7.3 ± 3.5^b^

a,b Means ± SEM (expressed in logarithmic scale) with distinct superscripts in rows differ by at least P < 0.05.

Sperm motility and the integrity of the sperm membrane and acrosome after thawing were greater after the HACCP implementation (P < 0.0001) than during the previous period ( Table 2 ).

**Table 2:** Post-thawing bull sperm quality, before and after eight months of the implementation of a HACCP system ^*^ .

Parameter (%)	Pre-implementation (n = 35)	Post-implementation (n = 21)
Progressive motility	33.7 ± 1.3^a^	57.7 ± 1.3^b^
Membrane integrity	47.9 ± 1.6^a^	85.3 ± 1.4^b^
Acrosome integrity	61.4 ± 3.0^a^	81.6 ± 3.6^b^

a,b Means ± SEM with distinct superscripts in rows differ by at least P < 0.0001.

* Comparisons by Wilcoxon ranks sum test for nonparametric data.

After the HACCP implementation, rejection of semen batches and doses were reduced (P < 0.01) by 12 and 10 percent points, respectively, in comparison with the previous period ( Table 3 ). During the four months prior to the HACCP implementation, losses due to opportunity costs were equal to US$704,290.00, whereas such losses were equal to US$274,740.00 during the period evaluated after the HACCP implementation.

**Table 3:** Semen production and opportunity cost during four months in a bull semen production center, before and after HACCP system implementation (October, November, December and January of two consecutive years).

Parameter	Pre-implementation					Post-implementation			
	Total	Rejected	(%)	Opportunity cost (US$) ^*^		Total	Rejected	(%)	Opportunity cost (US$) ^*^
Ejaculates	2,916	1,039	35.0	-		2,117	731	34.5	-
Batches	1,197	209	17.5^A^	-		944	46	4.9^B^	-
Doses	407,766	70,429	17.3^A^	704,290.00		378,520	27,474	7.3^B^	274,740.00

A,B Distinct superscripts in rows indicate difference of at least P < 0.01.

* Assuming a total cost of US$1.00 per semen dose produced and an average sales price of US$ 10.00 per dose.

## Discussion

This is the first study to report a positive impact of implementing a HACCP system specifically developed for a commercial bull SCPC, even though decision-support systems of such nature have been used in the food industry ( Ropkins and Beck, 2000 ; Lupin *et al*., 2010 ; Wang *et al*., 2010 ) and some of its concepts have been applied to compare microbiological contamination across boar studs ( Schulze *et al*., 2015 ). The results of the present study indicate that the combined use of preventive measures, periodical audit and corrective actions established through the HACCP system is truly more efficient than restricting quality control to inspection of the final product ( Ropkins and Beck, 2000 ). The improved quality control likely allowed the maintenance of the health status of the bulls housed in the SCPC and the hygienic collection, processing and storage of semen, which could result in negligible risk of infection for others animals or humans. Subsequently to the HACCP implementation, frozen semen doses produced in that SCPC presented improved sperm quality and reduced microbiological contamination, which resulted in greater financial revenues.

As the frequency of rejected ejaculates was unaltered compared to the previous period, the HACCP implementation apparently did not interfere on inherent bull characteristics, which reflects the fact that no relevant changes occurred in the bull’s inventory and genetic pattern. During the four months prior to the HACCP implementation, more than 70,000 frozen semen doses were discharged (17.3% of the total), leading to losses due to opportunity costs greater than US$700,000.00. Assuming the same rejection rates observed in the pre-implantation period and considering adjustments for the observed production with no HACCP system in place, losses during the second evaluated period would be US$ 65,377.65 and opportunity costs would add to US$ 653,376.55. Nonetheless, according to our simulation, with the implementation of the HACCP system, losses would be reduced to US$ 27,474.00 and opportunity costs would be equal to US$ 274,740.00. As most semen samples were discharged due to either reduced sperm motility or increase in abnormal sperm morphology, the changes introduced in the production process of the SCPC with the HACCP implementation not only led to an overall improvement in the quality of frozen semen samples, but also would result in considerable financial savings (more than US$400,000.00), as also observed in the fish industry ( Lupin *et al*., 2010 ).

Excessive bacterial in load semen may result in reduction in sperm motility and viability ( Althouse, 2008 ; Bussalleu *et al*., 2011 ; Sepúlveda *et al*., 2014 ), eventually leading to uterine infection in the inseminated cows ( Kaproth and Krick, 2004 ). Hence, semen doses produced in SCPC should have the lowest possible contamination ( Hueston and Sivula, 1988 ), which should be less than 5,000 CFU/ml according to the OIE (2014). However, due to the presence of microorganisms in the external genitalia and in the prepuce of bulls ( Thibier and Guerin, 2000 ) and to the possibility of further contamination during semen collection and processing ( Kaproth and Krick, 2004 ), collection of sterile ejaculates is virtually unfeasible. The health status of bulls and teaser cows was identified as a critical control point ( Goularte *et al*., 2015 ), because no subsequent step of semen processing could control the risk of disease transmission, if potentially pathogenic agents are present. However, after the HACCP implementation, routine semen collection procedures were standardized, including the toilet of bull's prepuce (preputial washing and prepuce hygiene – routine hygienic bull management) and the assembly of artificial vaginas, which likely reflected positively on decreasing microbial counts in fresh semen samples. For instance, prior to the HACCP implementation, the saline solution used to wash the prepuce was kept outdoors for 2-3 days in a container that was cleaned twice per week and the hygiene of the prepuce was done for all bulls before the semen collection procedures. After the HACCP implementation, a stock washing solution was kept at a refrigerator, the solution used to wash the prepuce was renewed on an everyday basis, the container was washed every time it was used, and the prepuce of each bull was sanitized prior to each semen collection.

Surprisingly, the number of CFU/ml in samples of cooled semen following the HACCP implementation was greater than during the previous period (4 months). Routine audit of the control points ( Goularte *et al*., 2015 ) detected that a tylosin batch included in the extenders was ineffective, allowing the growth of intrinsic contamination of the semen, which was reflected in higher CFU/ml in cooled semen. Nonetheless, after the discharge of such batch, extenders subsequently prepared with effective tylosin batches presented dramatically reduced counts of CFU/ml (Goularte *et al. *, 2017; ReproPel, Faculdade de Veterinária, Universidade Federal de Pelotas, Pelotas, RS, Brazil; unpublished data). Although, the microbial counts in thawed semen doses (final product) after the HACCP implementation were still above those recommended by the OIE (2014), such counts were lower than those observed during the previous period, due to changes in procedures adopted after the HACCP implementation. Before the HACCP implementation, the FT were washed, sterilized and used again, which was proven inefficient, as indicated by the high counts of CFU/ml observed in the FT. After the HACCP implementation, the FT were no longer reused and such contamination source was extinguished. Assuming that HACCP systems become more common in SCPC, the role of antibiotics in freezing extenders should be further investigated. A study with ram sperm suggested that when semen collection occurs with acceptable hygiene, it may be unnecessary to add antibiotics to extenders because the freezing-thawing process by itself might reduce the bacterial population up to levels accepted by international regulatory agencies ( Madeira *et al*., 2014 ). Thus, such findings may also explain the reduction in CFU/ml observed in frozen semen after HACCP implementation.

The HACCP implementation in the SCPC was beneficial for semen quality, as indicated by the improved sperm motility, membrane integrity and acrosome integrity observed in thawed semen samples. Such improvement may be related to the decreased microbial counts in thawed semen samples, since excessive microbial contamination reduces sperm viability ( Althouse, 2008 ; Bussalleu *et al*., 2011 ; Sepúlveda *et al*., 2014 ), mainly by apoptosis induction and necrosis, which can be partially responsible by reduction in sperm motility ( Moretti *et al*., 2009 ). Moreover, some pathogenic agents could directly affect bull fertility by causing reproductive diseases or damages in spermatozoa, preventing fertilization ( Givens and Marley, 2008 ). As observed for cooled boar sperm, increased *E. Coli* concentration in semen was associated with subsequent reduction in litter size ( Maroto Martín *et al*., 2010 ). Although analyses using either light or fluorescence microscopy were traditionally the standards for semen quality evaluation, currently many SCPC use multiparametric models that combine various *in vitro* assays, such as CASA and flow cytometry, that allow quick and precise analyses of a large number of spermatozoa ( Gadea *et al*., 2004 ; Broekhuijse *et al*., 2011 ; Vincent *et al*., 2012 ). As such analyses become more common in SCPC using HACCP systems, monitoring of the quality of sperm doses would likely be further improved.

Considering the fairly long timespan evaluated in the present study, some factors might potentially have influenced the responses observed subsequently to the HACCP implementation. Although the bulls evaluated in the previous period were not all the same evaluated subsequently, changes in the bull population dynamics during the period were minimal. Also, any bulls suspected to have health problems according to the serological monitoring routinely conducted in the SCPC (even before the HACCP implementation) were not used for semen collection. Therefore, the potential impact of the bulls for the quality of sperm doses cannot be neglected, but it certainly was kept under controllable levels by the preventive health procedures conducted during the period of interest. Furthermore, all measures designed to prevent errors on critical control points identified during the surveillance period conducted prior to the HACCP implementation ( Goularte *et al*., 2015 ) were audited six months later in a workshop conducted with the SCPC staff and management, allowing any deviation to be identified and corrected. Moreover, no staff turnover occurred during the whole training and audit period, indicating the HACCP implementation contributed to qualify the SCPC workforce and that the standardized procedures can be customized to be executed by distinct crews in other SCPC.

In conclusion, the implementation of the HACCP system in a bull semen production center was cost-effective, allowing reduction in financial losses due to opportunity costs. Those benefits were related to the identification of critical points in the production process, resulting in decreased microbial contamination, less rejection of batches and semen doses and improved semen quality after thawing.
